# RNA-Associated Co-expression Network Identifies Novel Biomarkers for Digestive System Cancer

**DOI:** 10.3389/fgene.2021.659788

**Published:** 2021-03-26

**Authors:** Zheng Chen, Zijie Shen, Zilong Zhang, Da Zhao, Lei Xu, Lijun Zhang

**Affiliations:** ^1^School of Applied Chemistry and Biological Technology, Shenzhen Polytechnic, Shenzhen, China; ^2^Institute of Fundamental and Frontier Sciences, University of Electronic Science and Technology of China, Chengdu, China; ^3^School of Electronic and Communication Engineering, Shenzhen Polytechnic, Shenzhen, China

**Keywords:** cancer, long non-coding RNA, WGCNA, MicroRNA, hub genes

## Abstract

Cancers of the digestive system are malignant diseases. Our study focused on colon cancer, esophageal cancer (ESCC), rectal cancer, gastric cancer (GC), and rectosigmoid junction cancer to identify possible biomarkers for these diseases. The transcriptome data were downloaded from the TCGA database (The Cancer Genome Atlas Program), and a network was constructed using the WGCNA algorithm. Two significant modules were found, and coexpression networks were constructed. CytoHubba was used to identify hub genes of the two networks. GO analysis suggested that the network genes were involved in metabolic processes, biological regulation, and membrane and protein binding. KEGG analysis indicated that the significant pathways were the calcium signaling pathway, fatty acid biosynthesis, and pathways in cancer and insulin resistance. Some of the most significant hub genes were *hsa-let-7b-3p*, *hsa-miR-378a-5p*, *hsa-miR-26a-5p*, *hsa-miR-382-5p*, and *hsa-miR-29b-2-5p* and *SECISBP2 L*, *NCOA1*, *HERC1*, *HIPK3*, and *MBNL1*, respectively. These genes were predicted to be associated with the tumor prognostic reference for this patient population.

## Introduction

Cancer is one of the most common and lethal disease types in humans, and the molecular mechanisms governing cancer progression have not been elucidated to date (Liang et al., 2019; Yang et al., 2020; Zhang Z.M. et al., 2020). Tumors of the digestive system are a serious problem for human health, and esophageal tumors, colorectal tumors, gastric tumors and rectosigmoid-junction tumors are among the ten most common cancers worldwide. These cancers cause the most deaths, and the incidence rate is increasing year by year (Fitzmaurice et al., 2017, 2018, 2019; Bray et al., 2018; Doja et al., 2020; Hong et al., 2020; Li S. et al., 2020; Zeng et al., 2020a; Zhang L. et al., 2020). Therefore, the study of digestive system tumors has important practical significance.

Digestive system tumors are a common malignancy in the clinic. Colorectal cancer is the combination of colon and rectal cancer cases (Slattery et al., 2007; Ghosh and Yan, 2020). Colorectal cancer ranks third in men and second in women in the rankings of cancer incidence and is commonly diagnosed despite the increased death risk worldwide (Torre et al., 2015; Ozkan et al., 2019; Li J. et al., 2020). Colorectal cancer (CRC) is derived from the accumulation of epigenetic and genetic changes that lead to cancer-related deaths worldwide. With significant advancements in the early diagnosis of colorectal cancer, the cancer mortality rate has increased in the United States (Rahman et al., 2015; Callahan et al., 2019; Ma et al., 2020). In European countries, colorectal cancer also has a high incidence, and the annual rate is increasing worldwide (Ferlay et al., 2015; Amer et al., 2020). Although the cancer-related death rate increases every year, treatments can be administered at the early stages of cancer (Pawa et al., 2011; Zhang Y. et al., 2019). Other digestive system tumors, such as esophageal cancer (ESCC), have been reported as the eighth most common cancer worldwide with a high cancer-related death rate (Ferlay et al., 2010; Abbas and Krasna, 2017). According to previous studies, almost 400,000 patients die from this disease among 450,000 diagnosed patients, the cancer-related death rate is approximately equal to 87.8% worldwide, and the 5-year survival rate is as low as approximately 25% (Rustgi and El-Serag, 2014, 2015). The most common cancer in Asia is gastric cancer (GC), which is the fifth ranking cancer leading to the third highest cancer-related death rate in the world. At stages I and IIA, the GC 5-year survival rate is 81.8–93.6%, but at stage IIIC, it is very low at only 17.9% (Amin et al., 2017; Lin et al., 2019).

Recently, network analysis has become a popular method for large-scale data (Cheng et al., 2019b; Zeng X. et al., 2019; Jin et al., 2020; Liu et al., 2020; Zeng et al., 2020b), and the WGCNA is a popular method for these coexpression network constructs (Jiang et al., 2010; Zeng W. et al., 2019; Iliopoulos et al., 2020). Five steps were included in this method: 1. a gene coexpression network was constructed by an adjacency matrix; 2. network modules were identified by the hierarchical cluster method; 3. modules with related phenotypes were analyzed; 4. module relationships were analyzed; and 5. key modules were found. The WGCNA method is widely used in biological networks and genomic data mining analysis, and also gives rise to network-based meta-analysis techniques (Langfelder et al., 2013).

The theoretical basis of the ceRNA (competitive endogenous RNA) hypothesis is that lncRNAs interact directly to regulate the expression of targeted genes and indirectly combine with miRNA sequences via the common miRNA response elements (MREs) of lncRNAs (Sardina et al., 2017). Cytoplasmic lncRNAs mainly affect mRNA reliability and translation mechanisms by binding miRNAs in the ceRNA regulatory network (Cao et al., 2018). Recently, analysis of lncRNA, miRNA, and mRNA networks has been identified to be involved in the progression of cancer (Zhang X. et al., 2017; Zeng et al., 2018), including endogenous cancer, hepatocellular carcinoma cancer and other malignant tumors, using the WGCNA method (Wang et al., 2010; Tay et al., 2014; Wei et al., 2014, 2018, 2019a,b; Thomson and Dinger, 2016; Jiang et al., 2018, 2019; Bai et al., 2019; Cheng et al., 2019a; Jin et al., 2019; Long et al., 2019; Shen et al., 2019). To date, there are few studies on digestive system malignant tumors. Therefore, integrated analysis of the regulatory functions of digestive system malignant tumors for lncRNA-miRNA-mRNA interaction networks requires large sample data and methods (Jiang et al., 2014; Zeng et al., 2016; Zou et al., 2016; Liu et al., 2017; Cheng et al., 2018; Cheng, 2019).

Competitive endogenous RNAs (ceRNAs) represent a novel gene expression regulation model and have attracted much attention from the academic community in recent years (Zhao et al., 2020). Compared with the miRNA regulation network, the ceRNA regulation network is more sophisticated and complex because the ceRNA regulation network includes more RNA molecules, such as mRNAs, pseudogenes, long non-coding RNAs (lncRNAs) and miRNAs. The ceRNA network provides a new perspective for transcriptomic and biological research.

In our studies, we used RNA-seq data from TCGA for free-scale gene coexpression network construction of digestive system malignant tumors. This study was able to provide novel biomarkers for these malignant tumors. Therefore, potential biomarkers were identified by using bioinformatics methods for integrative analysis based on the large amount of RNA-seq. According to the results of the analysis of malignant tumors, *NCOA1*, *HERC1*, *HIPK3*, *MBNL1*, *hsa-let-7b-3p*, *hsa-miR-378a-5p*, and *hsa-miR-26a-5p* were predictive or prognostic factors for malignant tumors.

## Data and Methods

### Date Collection

For data analysis in this study, gdc-client 1.5.0 ww^1^ and TCGAbiolinks 2.16.3 tools^2^ (Colaprico et al., 2016) were used from the TCGA database. A total of 437 samples of mRNA data, 388 samples of miRNA data and 385 samples of clinical data for colon cancer were obtained from TCGA, including colon cancer samples and normal samples. For colon cancer, there were over 500 individuals, including 480 tumor and 41 normal samples. Eighty-seven samples of mRNA data, 97 samples of miRNA data, and 87 samples of clinical data for ESCC were obtained from TCGA, including esophageal cancer (ESCC) samples and normal samples. There were 90 samples of mRNA data, 79 samples of miRNA data and 87 samples of clinical data for rectal cancer obtained from TCGA, including ESCC samples and normal samples. A total of 373 samples of mRNA data, 452 samples of miRNA data and 406 samples of clinical data for gastric cancer (GC) were obtained from TCGA, including ESCC samples and normal samples. Sixty-six samples of mRNA data, 63 samples of miRNA data and 63 samples of clinical data for rectosigmoid junction cancer were obtained from TCGA, including ESCC samples and normal samples. The guidelines of this study are from the TCGA website^3^.

### Computational Analysis of RNA-Seq Data

The level three data of these cancers from TCGA by Illumina HiSeq 2000 platform (Illumina Inc., San Diego, California, United States) including miRNA, lncRNA, and mRNA data were analyzed by Digital Gene Expression Data package (edgeR 3.30.3)^4^ and limma (limma 3.44.3)^5^ package in R. The genes were annotated gene symbols through the Ensembl database^6^ (Zerbino et al., 2018). According to these analyses, absolute log_2_(fold change) ≥ 2.0 and FDR ≤ 0.01 were set to screen the significant differentially expressed genes of mRNAs (DEGs), lncRNAs (DElncRNAs) and miRNAs (DEmiRNAs) between tumor tissues and normal tissues. Non-cancer-specific expression genes were filtered, and upregulated or downregulated genes were saved. The package ggplot2 3.3.2^7^ was used in RStudio to generate volcano plots of upregulated or downregulated genes. Meanwhile, the VennDiagram R package^8^ was used for the Venn diagram of the datasets.

### Construction of the Coexpression Network

The WGCNA package was used to analyze the data as described previously (Langfelder and Horvath, 2008). For the different conditions of tumor tissues, some samples may deviate from the actual situation. These tissue samples will affect the accuracy of the results. Therefore, these samples were removed before sample analysis. The function of goodSamplesGenes was used to verify if there were many missing values for each sample and to remove these samples from the total data. Then, the function hculst was used for cluster analysis of tissue samples. Appropriate soft thresholds need to be selected to build the network when the WGCNA method is performed. The soft threshold value should not only reach a scale-free fitting index of more than 0.9 but also requires greater mean connectivity of the network. Thus, 4 was selected for the soft threshold value. A good soft threshold is selected based on this rule. At the same time, the size of the gene module will be divided, and at least 30 genes will be identified for one module. If the correlation coefficient is greater than 0.75 for two modules, then these two modules will be merged into one module. To enhance the productivity of the modules, a cutoff (< 0.25) was selected to combine similar modules. After these modules were divided, the sample traits were associated with each module such that the most important associated modules for the traits were searched. Cytoscape 3.8.1 was used for the visualization of gene coexpression.

### Gene Ontology and Pathway Enrichment Analysis

The DAVID online tool was applied for the GO (Gene Ontology) and KEGG analysis and functional annotation of DEmRNAs. The DAVID^9^ database website was used for annotation and GO enrichment. KEGG pathways and GO terms enriched adopt adjusted P-value ≤ 0.05. WebGestalt^10^ is used for enrichment analysis that supports three well-established and complementary methods for enrichment analysis. The genes of the network were analyzed by the WebGestalt website. The R package of GOplot (version 1.0.2) was used for the GO analysis of the DEmRNAs of the five cancers.

### Hub Gene Identification and Validation

To identify the gene connectivity, Pearson’s correlation was used for the test. In general, hub genes existed in modules closely linked to traits (cor.geneTraitSignificance > 0.2), the modules with hub genes had high connectivity (cor.geneModuleMembership > 0.8), and the packages of cytoHubba and MCODE in Cytoscape 3.8.1 were used to search the hub genes.

## Results

### Identification and Validation of Differentially Expressed mRNAs, miRNAs, and lncRNAs

**FIGURE 1:**
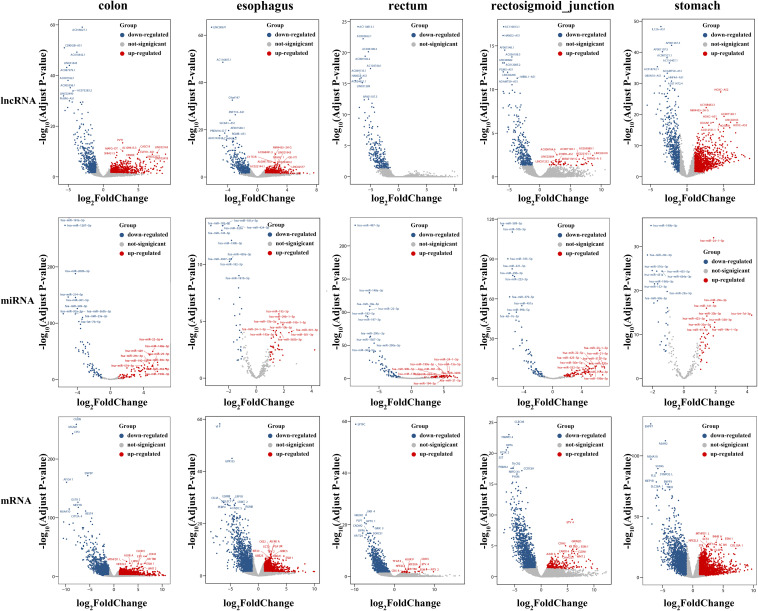
Volcano plots of DEmRNAs, DEmiRNAs and DElncRNAs in five cancers. The volcano map of differentially expressed lncRNAs (top), miRNAs (middle), and mRNAs (bottom). Red and blue spots represent significant up- and down-regulated RNAs, respectively. The remark for the gene symbols represent the significant up- and down-regulated RNAs.

**FIGURE 2:**
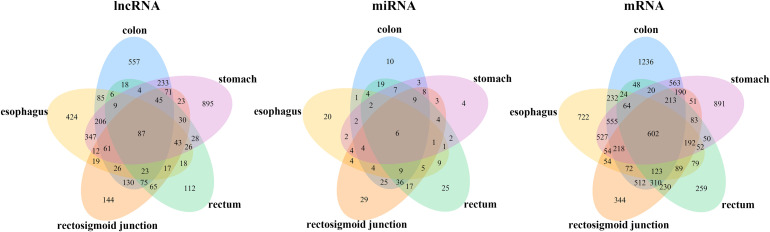
Venn diagram of the DEmRNAs, DEmiRNAs, and DElncRNAs in five cancers. Different colors indicate the different cancers. The numbers represent the common RNAs among different cancers. The color of blue, orange, pink, green and yellow represent for colon, rectosigmoid junction, stomach, rectum, and esophagus cancer, respectively.

To identify the differentially expressed mRNAs, lncRNAs and miRNAs, the standard of log_2_(fold change) ≥ 1 and q-value < 0.05 was used, and the EdgeR package of R (Law et al., 2016; Maza, 2016; Chen et al., 2017) was adopted to calculate separately for the normal samples and cancer samples of five cancers. For colon cancer, 4982 DEmRNAs, 149 DEmiRNAs and 1636 DElncRNAs were identified. There were 1409 differentially expressed genes in lncRNAs, 78 DEmiRNAs and 3659 differentially expressed mRNAs genes found between healthy and cancer-treated samples for esophageal cancer. A total of 3337 DEmRNAs, 168 DEmiRNAs and 871 DElncRNAs were identified between the rectosigmoid junction cancer tissues and matched normal control tissues; in addition, 2438 DEmRNAs, 156 DEmiRNAs and 607 DElncRNAs were identified for rectal cancer, and 4325 DEmRNAs, 62 DEmiRNAs and 2120 DElncRNAs were identified for gastric cancer compared with normal control samples. The distributions of differentially expressed DEmRNAs, DEmiRNAs and DElncRNAs were identified through volcano plots (Figure 1). A number of differences were found, and 87 DElncRNAs, 6 DEmiRNAs and 602 DEmRNAs were shared among the five cancers as shown in Figure 2.

### GO and KEGG Pathway Analysis of DEmRNAs

**FIGURE 3:**
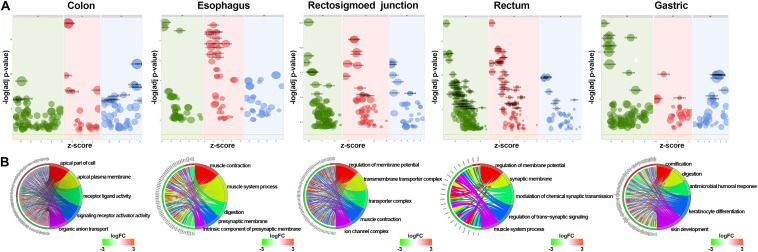
Identification of differentially expressed RNAs and GO enrichment of DEmRNAs. **(A)** Bubble plot of GO enrichment for cancers. Green represents biological processes; red represents cellular components; and blue represents molecular functions. **(B)** The top 5 significantly enriched GO pathways and relevant genes.

**FIGURE 4:**
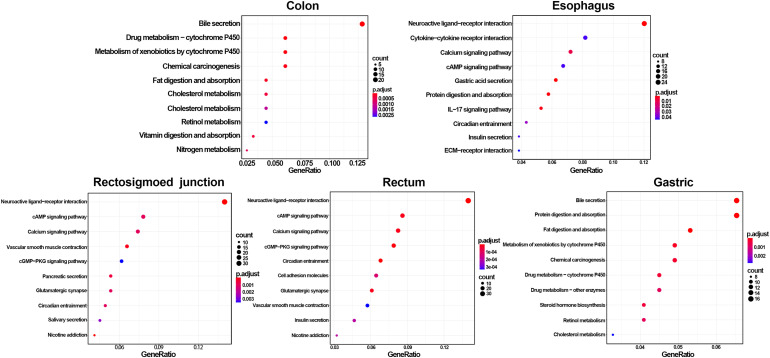
Identification of differentially expressed RNAs and KEGG enrichment of DEmRNAs. The color of the bubble represent the significant of p.adjust.

GO and KEGG methods were adopted to investigate the annotation of DEmRNAs for the five cancers (Figures 3, 4). Standards of log_2_(fold change) ≥ 3 and q-value < 0.01 were used for the analysis.

A total of 766 DEmRNAs were primarily enriched in biological processes (BP) such as organic anion transport for colon cancer (GO:0015711) and cell components (CC) such as the apical part of the cell (GO:0045177) and the apical plasma membrane (GO:0016324) as well as ligand activity (GO:0048018) and signaling receptor activator activity (GO:0030546) of molecular function (MF) receptor for colon cancer (Figure 3). In addition, KEGG enrichment analysis revealed that the significant pathways were “bile secretion,” “drug metabolism - cytochrome P450,” “chemical carcinogenesis,” “metabolism of xenobiotics by cytochrome P450” and “retinol metabolism,” which are associated with the progression of colon cancer (Figure 4).

To investigate the enrichment in esophageal cancer, 448 DEmRNAs were used and enriched in muscle contraction (GO:0006936), the muscle system process (GO:0003012), digestion (GO:0007586), the presynaptic membrane (GO:0042734), the intrinsic component of the presynaptic membrane (GO:0098889) of biological processes (BP) and cell components (CC) (Figure 3). In addition, “gastric acid secretion,” “neuroactive ligand-receptor interaction,” “protein digestion and absorption,” “IL-17 signaling pathway,” and “calcium signaling pathway” were the top 5 pathways of the KEGG analysis (Figure 4).

Rectosigmoid junction cancer was also analyzed by GO and KEGG enrichment; 623 DEmRNAs were screened out, and the top 5 GO enrichments were the regulation of membrane potential (GO:0042391), the transmembrane transporter complex (GO:1902495), the transporter complex (GO:1990351), muscle contraction (GO:0006936), and the ion channel complex (GO:0034702) of biological processes (BP) and cell components (CC) (Figure 3). The top 5 KEGG pathways were “neuroactive ligand-receptor interaction,” “nicotine addiction,” “vascular smooth muscle contraction,” “pancreatic secretion,” and “cAMP signaling pathway” (Figure 4).

Rectal cancer was also analyzed by GO and KEGG enrichment; 691 DEmRNAs were screened out, and the top 5 GO enrichments were the regulation of membrane potential (GO:0042391), the regulation of transsynaptic signaling (GO:0099177), the synaptic membrane (GO:0097060), the modulation of chemical synaptic transmission (GO:0050804), and the muscle system process (GO:0003012) of biological processes (BP) and cell components (CC) (Figure 3). The top 5 KEGG pathways were “neuroactive ligand-receptor interaction,” “circadian entrainment,” “cGMP-PKG signaling pathway,” “glutamatergic synapse” and “cAMP signaling pathway” (Figure 4).

Gastric cancer was also subjected to GO and KEGG enrichment analyses. A total of 637 DEmRNAs were screened out, and the top 5 GO enrichments were cornification (GO:0070268), digestion (GO:0007586), antimicrobial humoral response (GO:0019730) (Ji et al., 2019; Nadia and Ramana, 2020), keratinocyte differentiation (GO:0030216), and skin development (GO:0043588) of biological processes (BP) (Figure 3). The top 5 KEGG pathways were “Protein digestion and absorption,” “Bile secretion,” “Fat digestion and absorption,” “Chemical carcinogenesis,” and “Metabolism of xenobiotics by cytochrome P450” (Figure 4).

### Construction of the WGCNA Network

**FIGURE 5:**
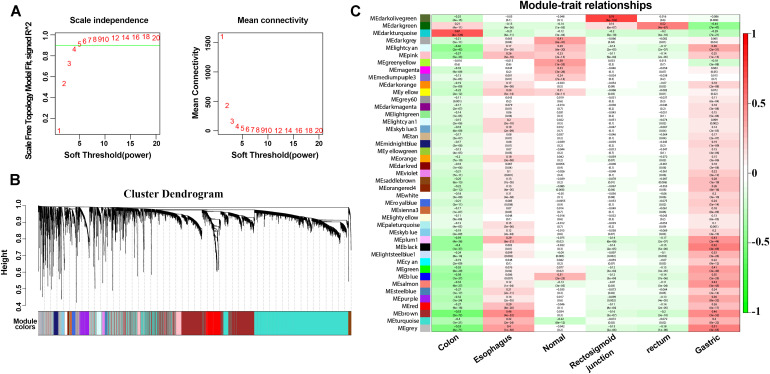
WGCNA method and module identification of the cancers. **(A)** Scale independence and mean connectivity of soft-thresholding values (β). **(B)** Cluster dendrogram of filtered genes. **(C)** Correlation between the cancers and MEs.

To explore further the key biological genes related to cancers, the WGCNA method was applied to select the DEmRNAs, DEmiRNAs and DElncRNAs. For further analysis, a soft threshold (β = 4) was adopted to guarantee a scale-free network with high scale independence and low mean connectivity (near 0) (Figure 5A). DEGs from the five cancers were divided into several modules by cluster analysis (Figure 5B). Approximately 43 modules were generated for the five cancers. The module trait relationship is shown in Figure 5C. The dark green module related to the colon, rectosigmoid junction and rectal tumors were the deepest (cor = 0.21, 0.16, and 0.52 P = 4E-11, 6e-07, and 4e-67). For the other two cancers, the esophageal and gastric cancers, the brown module was chosen according to the correlation (cor = 0.46 and 0.46 P = 4E-52, 3e-52). Dark green and brown modules were selected for further analysis (Figure 5C). The high correlation and high P-value indicated that these modules are suitable for hub gene identification in these cancers.

### Identification of the Hub Genes in the Five Cancers

**FIGURE 6:**
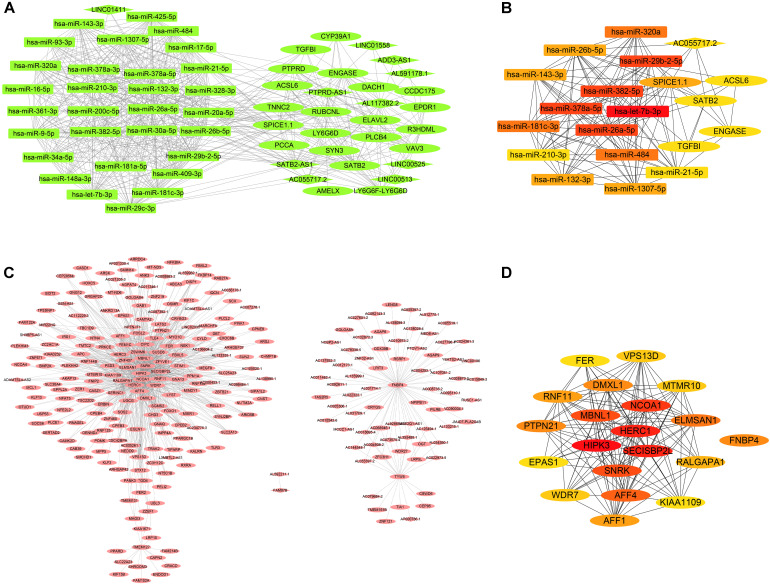
Coexpression network and hub gene network. **(A)** The significant gene coexpression network of colon cancer, rectosigmoid junction cancer, and rectal cancer in the dark green module. **(B)** The most significant hub gene network generated by CytoHubba from the dark green module. **(C)** The significant gene coexpression network of esophageal cancer and gastric cancer in the brown module, which has 1027 edges and 279 nodes. **(D)** The most significant hub gene network generated by CytoHubba from the brown module.

The dark green module was selected for the edges signifying the correlations in colon cancer, rectosigmoid junction cancer and rectal cancer with the filter criterion of a weight value greater than 0.02 by the WGCNA algorithm. A total of 629 edges and 61 nodes were obtained and input into Cytoscape 3.8.1 (Figure 6A). A network was generated by Cytoscape software, and the hub genes were obtained from the network by applying CytoHubba. The most significant hub gene network was discovered by CytoHubba, as shown in Figure 6B. The identified hub miRNAs included *hsa-let-7b-3p*, *hsa-miR-378a-5p*, *hsa-miR-26a-5p*, *hsa-miR-382-5p*, and *hsa-miR-29b-2-5p*. In addition, the brown module was selected for the edges signifying the correlations in esophageal cancer and gastric cancer with the filter criterion of a weight value greater than 0.12 by the WGCNA algorithm. A total of 1027 edges and 279 nodes were obtained and input into Cytoscape 3.8.1 (Figure 6C). The most significant hub genes were discovered by CytoHubba as shown in Figure 6D. The identified hub genes were *SECISBP2 L*, *NCOA1*, *HERC1*, *HIPK3*, and *MBNL1*.

We discover that most of the genes in dark green module that were significantly correlated with colon cancer, rectosigmoid junction cancer and rectal cancer were miRNAs. This dominance of miRNAs in dark green module suggests the possibility that lncRNAs play a significant role in these cancers through regulation of coding genes in key pathways. miRNA can act by guiding histone modifiers and chromatin modifiers to regulate transcription and play crucial roles in cell differentiation that ultimately determine cell fate.

### Functional Annotation of the Module of Interest and the Network Genes

**FIGURE 7:**
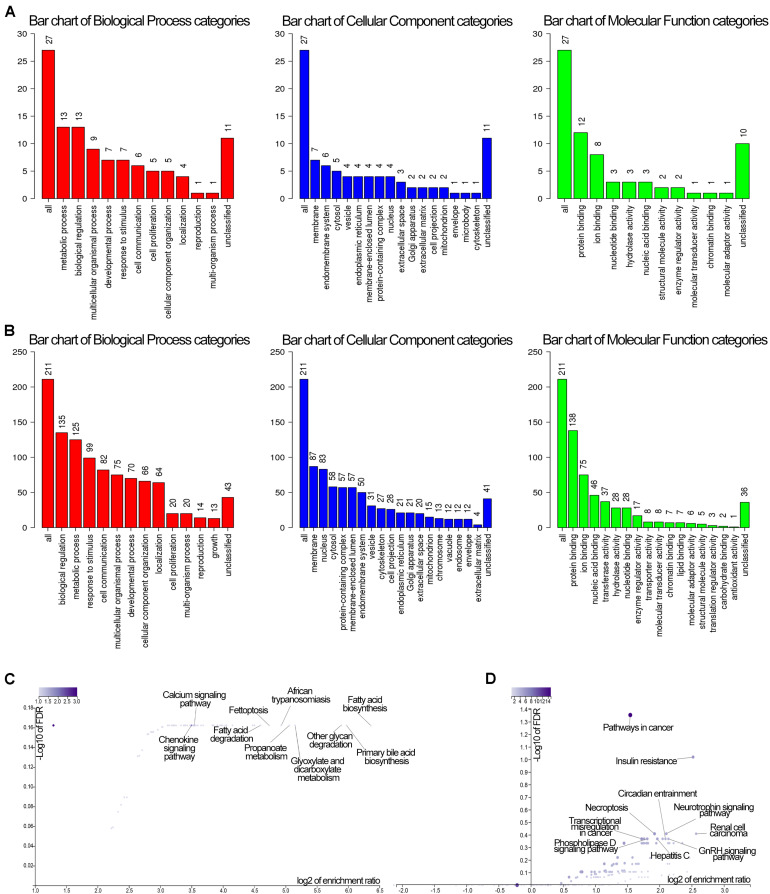
GO and KEGG enrichment of genes in the dark green and brown modules. **(A)** GO enrichment of the darkgreen module. Red color stand for biological process, blue stand for cellular component, while green stand for molecular function. **(B)** GO enrichment of the brown module. Red color stand for biological process, blue stand for cellular component, while green stand for molecular function. **(C)** KEGG pathway of the dark green module. **(D)** KEGG pathway of the brown module. The GO enrichment vertical axis suggests the number of enriched genes.

GO and KEGG analyses of the two modules of dark green and brown with 61 and 279 genes, respectively, in WebGestalt are shown in Figure 7. These were involved in molecular and functional biological processes and cellular components. For the dark green module, the genes were associated with metabolic process, membrane and protein binding, membrane among others (Figure 7A), and the genes in the brown module were enriched in biological regulation, membrane, and protein binding among others (Figure 7B). Analysis of KEGG pathways for the dark green module, the calcium signaling pathway, fatty acid biosynthesis and the chemokine signaling pathway were significant among all the pathways (Figure 7C), and pathways in cancer, insulin resistance and renal cell carcinoma were significant pathways in the brown module (Figure 7D).

## Discussion

Cancer of the digestive system is a common cancer worldwide. Recently, RNAs have been reported to be related to cancers. The WGCNA method was applied to analyze the related modules with different tumors and to identify the important genes of these tumors.

ESCC remains a serious burden on the health system worldwide, and some research has suggested that RNAs are associated with ESCC. For example, 2,046 circRNAs were frequently altered in ESCC tissues (Song et al., 2019). Several induced circRNAs were identified in radioresistant ESCC cells compared to normal ESCC cells (Su et al., 2016). Eight lncRNAs could be practical and reliable prognostic tools for esophageal cancers (Li W. et al., 2020). Additionally, the special functions of RNAs have been clearly demonstrated. For the analysis in gastric cancer (GC), 9 miRNAs, 41 lncRNAs, and 10 mRNAs were suggested and significant for GC (Pan et al., 2019). In addition, 15 lncRNAs were identified to be significantly related to the clinical features of colon cancer (Qian et al., 2019). In rectal cancer, COL1A1 and MZB1, as the key genes of rectal cancer, can interact with other genes correlated with shorter survival for patients (Wu et al., 2019). However, these studies presented research on only one cancer for the digestion system. Therefore, there is still an urgent need to identify the hub genes in digestive system cancers.

In this study, data from the TCGA website were adopted, and DEmRNAs, 168 DEmiRNAs, and 871 DElncRNAs were identified for the different tumors with the RNA-sequencing method and integrated bioinformatics analysis. Based on the WGCNA algorithm, the modules associated with different tumors were generated under the appropriate thresholds. Therefore, a network related to the different cancers was constructed in this study. Based on the network analysis, two modules, dark green and brown, were selected for further research. CytoHubba from Cytoscape 3.8.1 was used for this analysis, and several hub genes were chosen for the prediction.

Twenty hub genes were associated with the staging of esophageal cancer and gastric cancer, such as *SECISBP2 L*, *NCOA1*, *HERC1*, *HIPK3*, and *MBNL1*. *SECISBP2 L*, which encodes the SECIS binding protein 2 (SBP2), is a selenoprotein that exists in yeast, fungi and higher plants (Donovan and Copeland, 2009). However, the molecular mechanism of this protein has not been elucidated. *NCOA1* is nuclear receptor coactivator 1 (also known as SRC-1) belonging to the SRC gene family (Oñate et al., 1995; Xu et al., 2009). The abnormal expression level of SRCs has triggered cancers, such as breast, endometrial and ovarian cancers. The *SRC-3* and *SRC-1* genes have high expression levels in breast cancer. *SRC-1* regulates polyoma enhancer activator 3 (PEA3) and promotes breast-to-lung metastasis via transcription factors (Redmond et al., 2009; Walsh et al., 2012; Qin et al., 2015). HERC1 is a HECT and RLD domain containing E3 ubiquitin protein ligase family member 1 in humans. E3 ubiquitin ligases play a critical role in catalyzing ubiquitin transfer from E2 enzymes to the substrate in the ubiquitylation system. Similar to most E3 ubiquitin ligases, HERC1 has been shown to be a potential target for cancer therapy (Schneider et al., 2018; Dong et al., 2020). Homeodomain-interacting protein kinase 3 (*HIPK3*) is encoded by the *HIPK3* gene in humans. In NSCLC tissues, low expression of *HIPK3* was associated with poor survival rates. Therefore, *HIPK3* is considered a valuable biomarker for the survival of NSCLC patients (Liu et al., 2018). Muscleblind-like splicing regulator 1 (MBNL1), encoded by the *MBNL1* gene, is an RNA splicing protein and essential for MLL-rearranged leukemia cell growth (Itskovich et al., 2020). The isoforms of the MBNL1 protein affect cancer development and are targets for drug development (Li Z. et al., 2020). The results of the network indicated that these genes are also related to esophageal cancer and gastric cancer, and further research is necessary to determine the molecular mechanisms of these genes.

We eventually obtained 20 miRNAs associated with the staging of esophageal cancer and gastric cancer (Jiang et al., 2009), including *hsa-let-7b-3p*, *hsa-miR-378a-5p*, *hsa-miR-26a-5p*, *hsa-miR-382-5p*, and *hsa-miR-29b-2-5p*. Previous studies indicate that gastric cancer stem-like cells are derived from MKN-45- by the expression change of *hsa-let-7b-3p* (Salehi et al., 2018). Moreover, *microRNA-378a-5p* increased the expression level in melanoma cells and is a novel positive regulator of melanoma progression (Tang et al., 2018; Tupone et al., 2020). The expression of *hsa-miR-26a-5p* was downregulated in breast cancer tissues and appeared to have a poor prognosis for patients (Huang et al., 2019). However, overexpression *of hsa-miR-382-5p* increased oral squamous cell carcinoma cell invasion and migration (Sun et al., 2019). These studies suggested that miRNAs are also biomarkers for various cancers; however, the prediction of the interaction network indicated that these miRNAs are also related to colon cancer, rectosigmoid junction cancer and rectal cancer (Jiang et al., 2013).

## Data Availability Statement

Publicly available datasets were analyzed in this study. This data can be found here: https://www.cancer.gov/about-nci/organization/ccg/research/structural-genomics/tcga.

## Author Contributions

LX and LZ designed the research. ZC and ZS performed the research. ZC, ZS, ZZ, and DZ analyzed the data. ZC wrote the manuscript. All authors read and approved the manuscript.

## Conflict of Interest

The authors declare that the research was conducted in the absence of any commercial or financial relationships that could be construed as a potential conflict of interest.

## Footnotes

1 https://github.com/NCI-GDC/gdc-client/

2 http://www.bioconductor.org/packages/release/bioc/html/TCGAbiolinks.html

3 http://cancergenome.nih.gov/publications/publicationguidelines

4 http://www.bioconductor.org/packages/release/bioc/html/edgeR.html

5 http://www.bioconductor.org/packages/release/bioc/html/limma.html

6 https://uswest.ensembl.org/index.html

7 https://cloud.r-project.org/package=ggplot2

8 https://cran.r-project.org/web/packages/VennDiagram/

9 http://david.abcc.ncifcrf.gov/

10 http://www.webgestalt.org/
